# On the prospects of longtermism

**DOI:** 10.1111/bioe.13323

**Published:** 2024-06-18

**Authors:** Ingmar Persson, Julian Savulescu

**Affiliations:** ^1^ Oxford Uehiro Centre for Practical Ethics, Faculty of Philosophy University of Oxford Oxford UK; ^2^ Centre for Biomedical Ethics, Yong Loo Lin School of Medicine National University of Singapore Singapore Singapore

**Keywords:** easiness of harming, longtermism, moral enhancement, value lock‐in, William Macaskill

## Abstract

This article objects to two arguments that William MacAskill gives in *What We Owe the Future* in support of optimism about the prospects of longtermism, that is, the prospects of positively influencing the longterm future. First, it grants that he is right that, whereas humans sometimes benefit others as an end, they rarely harm them as an end, but argues that this bias towards positive motivation is counteracted by the fact that it is practically easier to harm than to benefit. For this greater easiness makes it likely both that accidental effects will be harmful rather than beneficial and that the means or side‐effects of the actions people perform with the aim of benefiting themselves and those close to them will tend to be harmful to others. Secondly, while our article agrees with him that values could lock‐in, it contends that the value of longtermism is unlikely to lock in as long as human beings have not been morally enhanced but remain partial in favor of themselves and those near and dear.

In his well‐argued and well‐researched book *What We Owe the Future* 1 William MacAskill ‘defends and explores the implications of longtermism, the view that positively influencing the long‐term future is one of the key moral priorities of our time’ (p. 253). He takes it that ‘there are two ways of positively influencing the long‐term future: by increasing ‘the average value of the future civilisation over its life span’ and by ‘increasing its life span’ (p. 254). Since he thinks it ‘most plausible’ that ‘wellbeing is all that matters, morally’ (p. 214) and that other sentient beings than humans count morally, an alternative formulation of his suggestion could be that an important moral aim is to see to it that the average well‐being of future sentient beings is as positive as possible and that there will exist in the future sentient beings with such a high average level of well‐being for as long as possible, ‘for millions or even billions of years to come’ (p. 27). For the sake of simplicity, we shall however restrict ourselves to considering human beings, but we share his view that animals count morally and that the ‘overall assessment of the lives of animals is … fairly pessimistic’ (p. 213).

To what extent is it probable that we shall realize this aim? MacAskill argues for an optimistic answer. He writes:

The key argument for optimism about the future concerns an asymmetry in the motivation of future people—namely, people sometimes produce good things just because the things are good, but people rarely produce bad things just because they are bad. (p. 218)

He suggests that ‘even the worst atrocities typically have been committed not simply because they are bad but as a side‐effect of other actions or as a means to some other end’ (p. 218). Even in the case of sadistic acts, ‘part of the motivation for these sadistic acts might have been to maintain power and signal status’ (p. 219), things that perpetrators see as good for themselves.

We are prepared to grant that there is an asymmetry with respect to people's motivation to produce what is good and bad, to the effect that they sometimes produce what is good simply for the reason that it is good but rarely, or even never, produce what is bad simply for the reason that it is bad.^2^ It would seem that something that is good in itself, like pleasure, cannot by itself sometimes produce a positive response in someone and sometimes a negative response. If there is such a variation in response to pleasure, it must surely be due to the influence of some property in conjunction with pleasure. It may of course be that neither the positive nor the negative response is produced by pleasure on its own, though as a matter fact it seems highly plausible that it by itself produces a positive response.

Granted that there is this motivational asymmetry—without which MacAskill's argument would break down—we do not see why it should result in that ‘eutopia is much more likely than anti‐eutopia’ (p. 220). Eutopia and anti‐eutopia are described as ‘the best and worst *possible* futures’ (p. 215), respectively. We doubt that any future could be the best or worst possible future in the strictest sense: that is, so good or bad that no better or worse future is conceivable. We could suggest a looser conception of eutopia and anti‐eutopia: as a pair of futures that we obtain if we make an equally strenuous effort to imagine an exceedingly good and an exceedingly bad future. But even granting that in something like this sense ‘eutopia is much more likely than anti‐eutopia’, it is hard to understand why this ‘gives us some reason to think that the expected value of the future is positive’ (p. 220). For it might still be the case that if we compare some slightly less good and bad futures, one of the bad ones will be most likely of all to be actualized.

Let us rather focus more directly on the question whether it is rational to believe that ‘the world will get better or worse in the long run’ (p. 215). And although it is not uncontroversial, let us assume, like MacAskill, that at present ‘most people have lives with positive wellbeing’ (2022, p. 201), so that a human future which is definitely better than the present is better than nonexistence.

Suppose it is true that when people intentionally or foreseeably produce what is bad, they do it as a means to some end which they think is good in some respect for someone or other, or as a side‐effect of such an end; then it may still be more probable that they produce a future which is worse than the present and on average worse than nonexistence in the longer run. This is because it is much easier to cause harm by disrupting well‐functioning systems, like living organisms or eco‐systems, than to maintain their good functioning. For their functioning well is dependent on many parts of them functioning well together, and if *any* of those parts is damaged, this may cause a breakdown of the functioning of the whole system. Thus, such systems are vulnerable. And since people are biased or partial towards themselves and those who are near and dear to them, there is a considerable risk that they disrupt the lives of others as means or side‐effects in their pursuit of smaller benefits to such favored parties.

To recycle an example we have used elsewhere to illustrate that it is easier to harm than to benefit: those of us who have access to a car and live in densely populated areas could any day single‐handedly kill or injure a great number of people by ploughing into a crowd, but we cannot go out any day and single‐handedly save the lives or heal the injuries of an equal number of people.^3^ Certainly, in exceptional circumstances—such as when we could prevent some terrorist from letting off a bomb that would kill hundreds—we may be able to save many lives, but these are exceptional circumstances of a kind in which most of us will never find ourselves and for the existence of which we have to rely on the actions of others. Such possibilities do not undermine our claim that it is *in general* much easier for us to harm than to benefit. Due to the fact that it is relatively easy to harm others, we are likely to be guilty of harming them now and then when we strive to fulfill our everyday self‐centered aims.

The fact that it is relatively easy to harm explains how human technology could have created weapons of mass destruction that could cause an enormous amount of harm but has not generated any instrument or equipment that could produce a comparable amount of benefits. MacAskill believes, reasonably, that ‘the risk of great‐power war in the next hundred years remains unacceptably high’ (p. 114). Such a war could involve the employment of nuclear weapons. He also believes that engineered pathogens could pose a significant risk to human survival (pp. 107–114). People could resort to using devastating nuclear or biological weapons in spite of their end not being to cause harm for its own sake but, for instance, being to attain ends like gaining more power or increasing their wealth.

Moreover, as MacAskill points out, ‘the badness of the worst possible world is much greater than the goodness of the best possible world’, for ‘the worst experiences that we can possibly feel are much worse than the best experiences that we can possibly feel’ (p. 216). The pain of the worst tortures—like that of the medieval English punishment of being drawn, hanged, and quartered—is more intense than the greatest pleasure and would not be voluntarily undergone to experience the latter. Fear could grow into terror or horror to which there is no counterpart as regards its opposite of hope or longing. Joy or elation can offer nothing to match chronic depression. Anger can be stoked up to fury and rage, whereas gratitude cannot be similarly intensified.^4^ This implies that individuals could suffer more when their bodies or property are damaged than they could enjoy their undisturbed preservation. Their great capacity for anger gives rise to a considerable risk for excessive vengeful action on those who cause them harm. MacAskill admits that this asymmetry with respect to feelings ‘gives grounds for pessimism’ (p. 216).

Pain is an example of something that is intrinsically bad or bad in itself for us, and pleasure is an example of something that is intrinsically good, or good in itself for us. But there is badness and goodness in a *comparative* sense; they could consist in the absence of what it is intrinsically good and intrinsically bad, respectively. This absence is evidently something that is *worse* than the presence of what is intrinsically good and *better* than the presence of what is intrinsically bad, respectively.

Now, as MacAskill remarks about something existing, ‘there is an asymmetry between preserving it and letting it be destroyed. If we preserve it and conclude later that it's not worth holding on to, then we can always change our minds. If we let it be destroyed, we can't ever get it back’ (p. 99). Imagine that we produce a life that is intrinsically good for the individual leading it. Then this state will not persist indefinitely; we must act both to keep the life running and to keep it good. However, if we stop it by painlessly killing the individual, we need not do anything more for this goodness to be absent forever.

If we are lucky enough to find ourselves with a well‐functioning organism, we still have to struggle to get a good life. This is because we constantly need resources to have a good life: adequate food and drink, comfortable shelter, medicine and treatment if we are ill or injured, enjoyable pastimes, and so on. But we do not have to make any efforts to have a bad life: if we do not do anything, we will suffer from thirst, hunger, cold or heat, and attacks from various adversaries and adversities. These factors will make our lives worse by damaging our bodies temporarily or permanently; they may even kill us, cause us to lose our lives entirely.

Loss of life is irreversible, and so are often loss of limbs and many damages to our bodies. Due to the longer duration that the badness consisting in the absence of something intrinsically good possesses, an action producing it will produce something comparatively bad that is greater than the goodness of an action preventing the disappearance of this intrinsic goodness. For this intrinsic goodness is likely to be ephemeral if it is not followed up by further efforts to sustain it.

Certainly, it is correspondingly true that an action producing goodness consisting in the absence of something intrinsically bad will produce a comparative goodness that is greater than the badness of an action that produces this intrinsic badness. Thus, if the world was a hellish place, it might be possible for us to produce a great amount of comparative goodness by painlessly extinguishing all sentient life. But this is irrelevant for present purposes, since we are now considering longtermism which is about ‘positively influencing the longterm future’ in the sense of the prospects of a future existence that is *better* than non‐existence. Such a world is harder to maintain than a world which is not better than non‐existence and which may be produced by extinguishing all sentient life. Such a world is also likely to be harder to maintain than a world that is *worse* than non‐existence, since we have seen that what is intrinsically bad is liable to be greater than what is intrinsically good, and it is easier to destroy what functions well than to keep it going.

Due to this greater easiness of harming, not only is the probability that people will cause overwhelming harm as a means or side‐effect of their biased ends considerable but also the probability that they will cause harm of such magnitudes *by mistake*. If there are many more ways in which things can go badly than well, it is much more probable that unintended or accidental effects will make things worse. Accidental and unforeseen effects are particularly likely to occur when we are dealing with advanced technology and the collective actions of huge numbers of people, since their effects are difficult to anticipate as they extend widely over the globe and far into the future. Such effects are especially scary with respect to weapons of mass destruction which, if they are not sufficient to bring about the extinction of the human species, could kill billions and cause such damage to civilization that life for the survivors is barely worth living. A simple mistake, and hell might break loose, but a paradise could not result as easily. The risks of very bad downfalls are much bigger than the chances of equally good windfalls.

MacAskill considers a future ‘consisting of an enormous number of people, spread out across the cosmos, living lives full of misery’ (p. 220). He finds it harder to explain how such a world could come about than a future ‘full of beings with long, blissful and flourishing lives’ if it is true that ‘Realistic dystopian scenarios are usually optimized for some other end, not to make the world as bad as possible’ (p. 220). But in contradistinction to the paradisical future the hellish future could result from mistakes, unforeseen side‐effects and misconceived means to other ends than to create hell for its own sake, as we have contended. Thus, though it is exceedingly unlikely that hellish scenarios are optimized with the end of making the world as bad as possible, it does not follow that such scenarios ‘seem very unlikely’ (2022, p. 220).

As long as we assume that overwhelmingly good and bad futures—eutopia and anti‐eutopia if you will—must be intentionally created for their own sake, we should indeed conclude that ‘eutopia is much more likely than anti‐eutopia’. But as we have argued, this is no longer so if we take into consideration the possibility that these futures come into existence unintentionally. This could happen because people are biased towards the closer future of certain smaller selected groups and are likely to cause greater harm to more people, especially in the more distant future, as a means or side‐effect of the former good. Such outcomes could easily spark violent conflicts. People might also easily produce such harm by mistake, perhaps not realizing how probable such harm is or how great its magnitude is because their desire for the good of favored parties makes them prone to engage in wishful thinking. Such harm is likely to be greater since it often consists of a removal of goods that is irreversible and the suffering that such a removal of what is good gives rise to is greater than the pleasure its possession yields.

MacAskill conjectures that even if human civilization suffers a major setback, it may be possible for it to recover under certain conditions—for example, if easily accessible fossil fuels have not been burnt through (p. 142)—but he nevertheless judges that the risk that human civilization will end this century is ‘far too high for us to be comfortable with’ (p. 142). Civilization ending in this fashion would be something immeasurably bad, both because of the amount of suffering it is likely to bring to humans (and other sentient beings)—remember that suffering can be more intense than happiness—and because it rules out worthwhile life for the rest of the future.

To be sure, it will not be the worst possible outcome, anti‐eutopia in the strictest sense, since it will not maximize suffering, but it will still undercut the longtermist aim of ‘positively influencing the longterm future’ in the present sense. Even if humans are not motivated to harm others for their own sake, this outcome is likely because humans are likely to harm others a lot as means or side‐effects of their pursuit of smaller benefits for favored parties, or accidentally by making mistakes in such pursuits, due to the fact that it is so much easier to harm than to benefit.

Imagine instead that civilization does not end but recovers. Then the recovery may not be sufficiently strong or long‐lasting to make up for the enormous suffering that the preceding devastation involved. In periods in which there is a serious shortage of resources, there is a considerable risk of new conflicts over the scarce resources, and these conflicts could undo what recovery has been achieved, so that the average of well‐being will never be anything like what it was.

It seems reasonable to think that if any of these catastrophic developments were in store for the future, it would be better if all human life was painlessly extinguished before they took off. (If the well‐being of nonhuman animals is also taken into consideration, this judgment is probably strengthened, as MacAskill conjectures [pp. 208–213].) But perhaps catastrophe could be avoided without such a drastic measure as annihilation of the human species or a more sophisticated civilization. He draws attention to the phenomenon of a *value lock‐in*: ‘an event that causes a single value system, or a set of value systems, to persist for an extremely long time’ (p. 78). He proposes that in ‘history as a whole’ values exhibit a dynamic of ‘early plasticity, later rigidity’ (p. 43). That is to say, during a certain period, it may have been an open question what attitude a community like early Christians will adopt to a certain practice like male circumcision or infanticide, but eventually its attitude was fixed for well over a millennium and became very hard to change. If values that support longtermism got locked in thus, this would clearly boost the prospects of a good future.

We must be careful about locking in values, however, for serious value mistakes could be locked in, as MacAskill recognizes: for instance, if dominant values of the eighteenth century were locked in, we would still have slavery and discrimination of women. However, the necessity of leaving room for values to improve gives rise to a ‘lock‐in paradox’: ‘We need to lock in some institutions and ideas in order to prevent a more thoroughgoing lock‐in of values’ (p. 101). In other words, we need to lock in the value of *tolerance*. But tolerance of divergent attitudes creates the risk that attitudes which are opposed to tolerance slip in under the radar. The result could be violent confrontations in which intolerant camps are victorious, and the more complete value lock‐in of totalitarian regimes with corrupt values are established.

However that may be, longtermism is unlikely to be a value that locks in globally. A necessary condition for this instance of lock‐in to occur is that this aim becomes a leading ideal worldwide, but this is unlikely to happen for the following reason: ‘It's hard for an abstract ideal, focused on generations of people whom we will never meet, to motivate us as more salient problems do’ (p. 5). It took MacAskill himself ‘a long time to come around to longtermism’ (p. 5); so, we should not be too hopeful about morally less motivated and well‐informed people to come around it. We should rather expect that people in general continue to manifest the kinds of partiality towards people close to them and towards the near future that create conflicts within societies and between societies and hamper the far‐reaching collective action required to tackle global problems for the future like anthropogenic climate change, the depletion of natural resources, and global inequality.

Therefore, we have argued in *Unfit for the Future* and other publications that moral enhancement, including bioenhancement, counteracting such partiality is necessary for safe‐guarding the future. In addition, perhaps also cognitive enhancement is necessary so as to make humans capable of predicting more accurately the consequences of their actions. This is also necessary for the policy of longtermism lock‐in. As the human power to cause harm continues to grow, and the risk that it will be used seemingly increases rather than decreases in view of the scarcity of resources that is likely to result from climate change and overexploitation, the threat of a major catastrophe seems greater than ever. The elephant in the room of the future is *Homo sapiens* who is in urgent need of moral enhancement and perhaps also cognitive enhancement, by whatever available means that are safe and effective.

## CONFLICT OF INTEREST STATEMENT

Julian Savulescu is a Partner Investigator on an Australian Research Council grant LP190100841 which involves industry partnership from Illumina. He does not personally receive any funds from Illumina. Julian Savulescu is a Bioethics Committee consultant for Bayer. Julian Savulescu is an Advisory Panel member for the Hevolution Foundation (2022‐) The other author declares no conflict of interest.

## Footnotes

1 London: Oneworld, 2022. Unprefixed page references in the text are to this book.

2 Cf. Persson, I. (2021). *Morality from compassion*. Oxford University Press, pp. 65–66.

3 Cf. Persson, I., & Savulescu, J. (2021) *Unfit for the future*, Oxford University Press, 12. See also Persson, I., & Savulescu, J., (2013). Getting moral enhancement right: The desirability of moral bioenhancement. *Bioethics*, *27*, 124–131.

4 Cf. Persson, op. cit. note 2, pp. 115–118.

